# Supplementary material for: Patterns of Intron Gain and Loss in Fungi
Source: PLoS Biol. 2004 Nov 30;2(12):e422. doi: 10.1371/journal.pbio.0020422 (PMC532390; doi:10.1371/journal.pbio.0020422)
Supplement: Table S1 — Also available at http://genes.mit.edu/NielsenEtAl/. (4.3 MB ZIP). [file pbio.0020422.st001.zip › NielsenEtAl/html/466.html]

AN4171.1.NCU03886.1.MG01047.1.FG08449.1


```
 CLUSTAL W (1.82) Multiple Sequence Alignments - Introns Inserted


Sequence 1: MG01047.1	519 aa
Sequence 2: FG08449.1	480 aa
Sequence 3: NCU03886.1	498 aa
Sequence 4: AN4171.1	521 aa
Alignment Length: 549 aa
Number Identitical Residues: 151 aa
Alignment Score (without introns) 8725


MG01047.1 	---MASPPEG--MSRATT------------SISDD-AVPENDPTG0TAGLLAERLSAWKH
NCU03886.1	----MSANASAAPSRQTTM---------MSNASDDTAIPEFDPNS0TAGILVQRLRAWKH
FG08449.1 	---MASPTTTNLPSRATTG---------FSTQSED-AIPEVDPSS0TAGLLAERLQAWKH
AN4171.1  	MSATSSPVEARLPTRSNTLRTVSTGTERRASLSDDEAIPGGDSNE0TTNLLVERLRAWKH
          	 ::  *.      :* .*  : ::.:.  :. *:* *:*  *..  *:.:*.:** ****

MG01047.1 	AVGYLEEFMVAVEKAHKNQAKEYEKVLK~SISKPLKEGHHFDQSLG---GVAGFFENMRV
NCU03886.1	AVAYLEDYVEAVEKIHHSQAKEYEKVLK0TISHPLREGDHFSQHATNVTGVAAMFEVLRA
FG08449.1 	AVGYIEEYMKAMERIHGHHAKEYEKALK0AISNPLKEGHHFDQSLG---GMAGFFENMRS
AN4171.1  	MCGYLEDYVSVTAKVQKGLSKDYEKVLK0TVNEPLKEGHHFSQSAG---GVASFFENIRA
          	  .*:*::: .  : :   :*:***.** ::..**:**.**.*      *:*.:** :* 

MG01047.1 	NTE~AMVNTNIETEKTIKGTVLPIIERLHKEIKHKTKELSHGAQKSAKDVEKARNVTQKH
NCU03886.1	NTQ~GMINSNLETQKKLKETVLHDLKGLHHEIKDYSKRLSHSLNKGAKAVEKSRNTTQKH
FG08449.1 	NTQ~SLINTNIETEKSIKGSVLPVLERLHKEIKHKAKELAHGAEKGAKEVEKARNTTQKQ
AN4171.1  	NTQ0GMINLYADGEKNLRNSVLPTLEKLHKEIKAKAKELQTGASKAAKAVEKARNVTQKH
          	**: .::*   : :*.:: :**  :: **:***  :*.*  . .*.** ***:**.***:

MG01047.1 	IELLGQQTAAFE-SSGAKLDPSNDPYVLKRGVLHRLNQQVMDENNHRNDLLSVQNNFQAF
NCU03886.1	IELLSQHVASFP-TSGADLNPADDPYVLRQGVIYRLHNQIQDENNHRTELITAQAQFMEF
FG08449.1 	IELLGQHSASFD-SAGGHLNSNDDPYVVYRGVLHRLNSQVIAENNHRNDLIAVQNNFHAF
AN4171.1  	IELLGQQTAALDSAAGNKLDTSHDPYIVRRGVIHRLNKQVIEENNNRQEIITVQNNFQQF
          	****.*: *:: :::* .*:. .***:: :**::**:.*:  ***:* ::::.* :*  *

MG01047.1 	EMHIVEVMQQAMEAFNQSAGGQAEKMRALYSDILGAAQRMPPDFEWKGFVQRNMENLIDP
NCU03886.1	EVKVVKSIQKAMEDLNTLAGGHAEHVRGLYAQVLQTSQNIPPDFEWQEFRVRSADVLVNE
FG08449.1 	ETHIIEVIQQAMEAFTQLAGGQADKTRALYNDQLSVIQRVAPDFEWNAFNARSGDRLVDP
AN4171.1  	EAHVLQTIQAAMEQLVVFITGQTERQKTLYSDILGNLQRIPAEFEWVNFMTRNASSLVDP
          	* :::: :* *** :     *:::: : ** : *   *.:..:***  *  *. . *:: 

MG01047.1 	NENPRTVDSIT--FPNMDHKGTKSLIEGSLERKSRNKLSWGWSTGYYVVTPSKFLHEFKD
NCU03886.1	NEPPRELGHLLPLVPNADNPSTKALIEGSLDRKSRNKLSWGYNTGYYVVTPARYLHEFKD
FG08449.1 	NEPARSVDAIQ--FPNMDHPATRALIEGSLERKSRNKLSWGYSTGYYVITPSKYLHEFKD
AN4171.1  	DAPPRSIANIN--FPNQDHPATQPLISGTLQRKSRMALK-GYSSFFYVATRARYLHEFKD
          	:  .* :  :   .** *: .*:.**.*:*:****  *. *:.: :** * :::******

MG01047.1 	SDNLRKDPVPELSIYLPDAVIGTPNMEK--FNVKGKDVSK-GISGKLAGSAELAFKAHTA
NCU03886.1	NDPKRKDPQPELSIYLPDAIIGAPKDDK--FNVKGKDKSK-GIGSKLAGTSELQFKAHSA
FG08449.1 	SDNNRVDPKPELSIYLGDAVIGTVQGEK--FSVKGKDKSK-TMSSKLTGSTELNFKAHTT
AN4171.1  	NDDFNRDPSPEISLYLPDCHIVSVDEVKHTFTIKGKDVSSNAIGNAFHTNTEFVFKAGSA
          	.*  . ** **:*:** *. * : .  * :*.:**** *.. :.. :  .:*: *** ::

MG01047.1 	ADADRWFEVIKSVAGATGAKRVNTDANAASAAAAAMSSPASPTSPTSP--TEKNAAQPLP
NCU03886.1	AEAQKWFDIIS---------------NVAGALGPGVATPVTTPTPDTE--DKKLEAATNN
FG08449.1 	ADAQKWFQVLS-------------ECGKATAPLSTGSSPAG-STPTSP--NPQVTADPLD
AN4171.1  	AEAKEWVNILR-----------DAAKAPIATTTAAAVSPSTVTSPTSPGAASTATAAPVQ
          	*:*..*.:::            .:      :  .   :*   .:* : .:     * .  

MG01047.1 	PLATDQPKQESGVTGADTVASPDALSPKSAQDSGLASPQTAGTTPQAVSPTTAEAPKPQT
NCU03886.1	LTLNEAQTLPPSPHETGVVASPINISPVDSKAAGTAAP----------------PAVSPA
FG08449.1 	------SKVQSPIT---------PVNEHSIQEAGVVG---------------GEAPVAAP
AN4171.1  	PPAYEKESGAGTPPAVSRTNTTATTASTAEKVPSPVSEKT-----------EATAAAPST
          	    .  .        .   :.        : .. .. .:            . .. . .

MG01047.1 	VVTDQKKPMDA-
NCU03886.1	AAVPEKGALPK-
FG08449.1 	AVAAEKKETAA-
AN4171.1  	AAAAEKAPLEKS
          	... :*     :
```
